# Effects of an Early Handling-Like Procedure and Individual Housing on Anxiety-Like Behavior in Adult C57BL/6J and DBA/2J Mice

**DOI:** 10.1371/journal.pone.0019058

**Published:** 2011-04-21

**Authors:** Timothy J. Flanigan, Melloni N. Cook

**Affiliations:** Department of Psychology, University of Memphis, Memphis, Tennessee, United States of America; University of Adelaide, Australia

## Abstract

Manipulations of rearing conditions have been used to examine the effects of early experience on adult behavior with varying results. Evidence suggests that postnatal days (PND) 15–21 are a time of particular susceptibility to environmental influences on anxiety-like behavior in mice. To examine this, we subjected C57BL/6J and DBA/2J mice to an early handling-like procedure. Pups were separated from dams from PND 12–20 for 30 minutes daily or received standard care. On PND 21, pups were weaned and either individually- or group- housed. On PND 60, anxiety-like behavior was examined on the elevated zero-maze. Although individually- housed animals took longer to enter an open quadrant of the maze, they spent more time in the open than group-housed animals. Additionally, we observed a trend of reduced anxiety-like behavior in C57BL/6J, but not DBA/2J mice that underwent the handling-like procedure.

## Introduction

There is a large and growing body of preclinical literature indicating that early-life experiences can permanently alter neurodevelopment as well as the activity and functioning of the hypothalamic-pituitary-adrenal (HPA) axis (for examples see: [1], [2]) resulting in either a compromised or enhanced ability of the organism to respond to stress. It is likely that both experiential and genetic factors, and their interactions, largely determine the nature of these responses [2]. A variety of manipulations have been used to investigate the effects of stressors in animals, and long-lasting effects on neuroanatomical structures, neurotransmitter system functioning, and behavior have been noted (for reviews see: [3]–[5]). It is evident from the clinical and preclinical literature that early-life experiences/manipulations influence the emergence of adult behavioral phenotypes including those anxiety- and drug-related. In fact, differences in emotionality noted during adulthood are likely shaped early in life [1].

Manipulations of an animal's rearing context have been commonly used to study the effects of early environment on a variety of phenotypes. Maternal separation (MS) and handling paradigms are among the best documented pre-weaning environmental manipulations. These procedures have generally been carried out during the first few weeks of life (generally PND1-14 or PND 1–21). Although some rodents undergo what has been termed a stress hyporesponsive period (SHRP) from approximately PND 4–14, during which they display little or no physiological response to stressors [6], manipulations applied during the SHRP can influence behaviors later in life (for review see: [7]). There is a limited number of reports on the effects of MS or handling following the SHRP in mice [8]. However, studies indicate that rearing conditions can alter development of the mouse's HPA axis responsiveness [9] and that changes in the neural systems mediating anxiety-like behaviors undergo critical development following the SHRP [10] and likely remain plastic during the pubescent and juvenile phases of mouse development [11].

Isolate- or individual-housing has also been used to examine the effects of environment on a variety of phenotypes. Variations of this procedure either house animals in individual cages or socially isolate them from other animals, often visually and/or acoustically. The reported effects of individual-housing on anxiety-like behavior have been diverse and have been studied more extensively in rats than in mice. For example, in elevated plus-maze (EPM) studies, an anxiogenic effect of individual-housing is commonly reported in rats [12]–[14], whereas in mice anxiolytic-like [15], [16] or null effects have been reported [17]. The limited availability and disparate results of reports on how individual-housing affects behavior in mice highlights the need for additional studies in reference populations.

The purpose of this experiment was to examine the effects of both an early-life handling-like procedure (EHLP), performed after the SHRP, and individual-housing on anxiety-related behavior in the adult mouse. We refer to our manipulation as an early handling–like procedure because it is akin to both MS and early-handling, but it does not fit the classical definitions of either. MS, as most commonly defined, separates the dam and the pups for 180 min while early-handling is generally defined by a 15 min separation (for example see: [18]). However, there is great variation in these procedures. For instance, ‘handling’ has been used to refer to a 60 s separation [19], and the term MS has been used to describe weaning at PND 14 [20]. Here, we separated dams from their pups daily for 30 min at PND 12–20, as considerable evidence indicates that PND 15–21 may be a time of particular sensitivity to environmental influences on anxiety-related behavior (for review see: [10]) yet few studies have examined such manipulations during this period. Based on what has been reported in the literature about the effects of other early life events, we expected the preweaning manipulation to increase anxiety-like behavior [21]. On the other hand, we expected the post-weaning manipulation to decrease anxiety-like behaviors. These expectations were largely based on the effects of similar manipulations on other behaviors including those drug-related [22], [23]. Like others [19], [24], we also sought to determine whether this pre-weaning EHLP interacts with a post-weaning manipulation (housing condition) to influence anxiety-like behavior; the expectation being that anxiogenic effects of the EHLP would be attenuated by the subsequent individual housing.We chose to examine these manipulations and their interactions in C57BL/6J (B6) and DBA/2J (D2) mice because they are among the most widely available and thoroughly phenotyped inbred strains, making them excellent reference populations. Further, B6 mice generally have lower corticosterone (CORT) secretion in response to stress [25], [26], lower quality maternal behavior [27], and lower levels of anxiety-like behavior [28], [29] than D2 mice. Based on these known differences, we expected that the B6 strain would be more sensitive to the proposed anxiogenic effects of the EHLP and effects to be less pronounced in the D2 strain. On the other hand we expected that, given the higher levels of anxiety in the D2 strain, this strain would be more sensitive to any anxiolytic effects of individual housing. Because sex differences in these behaviors have been understudied, we were also interested in the how this variable interacts with these manipulations.

## Method

### Ethics Statement

All experimental procedures and husbandry practices were approved by the University of Memphis Institutional Animal Care and Use Committee (Protocol No. 0303) and in accordance with the National Institutes of Health Guide for the Care and Use of Laboratory Animals.

### Subjects

Male and female B6 and D2 mice were purchased from the Jackson Laboratory (Bar Harbor, ME, USA) and put into breeding. To reduce unnecessary handling of animals beyond that of routine animal care, litters were not culled or sexed [9]. On PND 12, entire litters were either removed from their home cage for a period of 30 min or left undisturbed, outside of routine animal care. Similar to others [30], the EHLP involved first removing sires from the cages, followed by the dams, and finally the pups. The pups were placed in a clean cage with bedding and carried to a separate room in the animal facility (23°C) for the 30 minute period. We did not use any artificial heat sources, as body temperature loss in response to separation tends to decrease around PND 10 and body temperature tends to increase in response to handling at PND 15 [31]. After 30 min, the pups were returned to their home cage, followed by the dam and then the sire. The EHLP continued daily through PND 20. On PND 21, animals were weaned and group-housed (with same sex littermates in groups of two to five) or individually-housed. Individually-housed animals were kept in the same room in the animal facility as all other animals in the study. No attempts were made to isolate individually-housed animals with regard to visual, acoustic, or olfactory stimuli. Four groups resulted from our experimental design that we identify as follows: CON =  animal facility reared, group-housed; EHLP = handled, group-housed; IND =  animal facility reared, individually-housed; and EHLP+ IND =  handled, individually-housed. All animals had access to food and water *ad libitum* and were maintained on a 12:12 light/dark cycle with lights on at 06:30 and lights off at 18:30. The average temperature in the vivarium was 23°C and humidity ranged from 30–70%. All animals were tested in the elevated zero-maze (EZM) on PND 60.

### Behavioral Testing

Elevated zero mazes were manufactured by AccuScan Instruments (Columbus, OH, USA). The apparatus is an elevated black circular platform consisting of open and closed quadrants. Because we are interested in the avoidance of open versus closed areas and to minimize differences in light intensity between the open and closed quadrants, the closed quadrants are enclosed by clear acrylic walls 28.5 cm in height [32]. The closed quadrants are each equipped with infrared light beams allowing the amount of time spent and activity in the closed quadrants to be monitored. The open quadrants have a slightly raised Plexiglas lip to prevent the mice from falling off of the maze. The zero-maze has been described in detail elsewhere [28].

Mazes were separated from one another by solid partitions such that each maze was equidistant from three extra-maze walls. A greater range of behavior is generally displayed when testing is performed under dim and/or red light [33], [34]. Therefore, like others [35], each maze was dimly lit by a 15W red light bulb suspended approximately 125 cm above the maze, providing an average illumination of 14 lx at the level of each quadrant.

On the day of testing, animals were acclimated to the darkened holding area prior to testing. Test duration was five minutes. Animals were placed in a closed quadrant to begin the test period. Latency to enter an open quadrant, total time spent in open and closed quadrants, and activity in the closed quadrants were recorded. All mice entered an open quadrant of the maze. Activity levels can vary greatly because their measurement is a function of time spent in the closed quadrants; therefore, we evaluate activity as beam breaks per second spent in the closed quadrants. Mazes were cleaned with 70% isopropanol and allowed to dry between mice. All testing took place between 10:00 and 14:00.

### Statistics

To avoid litter effects, when more than one animal from a given litter was included in an experimental group, the mean for those subjects was treated as a single observation [36]. Thus, each observation in the analyses corresponds to a single litter. The *n* used for the analyses is reported in Table 1 along with the total number of animals tested in each condition.

**Table 1:** Observations used for analyses and number of animals tested.

	B6		D2	
Treatment	Male	Female	Male	Female
CON	8 (15)	11 (15)	3 (10)	5 (15)
EHLP	8 (15)	8 (15)	6 (14)	3 (11)
IND	9 (14)	7 (15)	8 (12)	8 (14)
EHLP + IND	7 (10)	5 (11)	7 (12)	8 (13)

*Note*. Numbers represent the number of observations used for analyses and correspond to unique litters used to form each treatment cell. Numbers in parenthesis are the actual number of animals contributing to each group. CON  =  not handled and group reared, EHLP  =  handled and group reared, IND  =  not handled and individually reared, EHLP + IND  =  handled and individually reared.

Data for each measure were analyzed using a four-way analysis of variance (ANOVA) using strain, sex, handling (i.e. underwent the EHLP or did not), and housing (i.e. individually or group housed) as between subject factors. Where significant interactions were found (*p*<.05) analysis of simple effects was employed. Effect sizes were calculated as described elsewhere [37]. Analyses were performed using SPSS 12.

## Results

### Latency to enter an open quadrant

Data for this variable violated the assumption of homogeneity of variance; therefore, a logarithmic transformation was performed. For ease of presentation; however, we present untransformed means and standard errors in Table 2. ANOVA revealed a significant effect of strain, *F* (1, 95) = 25.724, *p*<.001, with B6 mice entering an open quadrant sooner than D2 mice. There were no significant effects of sex or handling (*p*>.50, for both). However, a significant housing effect was found, *F* (1, 95) = 4.227, *p* = .043. Group-housed mice entered an open quadrant sooner than individually-housed mice. There were no significant interactions (*p*>.10, for all).

**Table 2:** Means and standard errors for latency to enter an open quadrant (s).

	B6		D2	
Treatment	Male	Female	Male	Female
CON	5.154(1.516)	3.559(.909)	9.654(1.008)	12.642(3.337)
EHLP	5.109(1.622)	4.763(1.107)	16.483(5.959)	10.864(4.828)
IND	9.157(2.114)	5.926(1.362)	16.738(4.302)	17.981(7.304)
EHLP + IND	7.338(2.784)	7.660(1.253)	19.733(2.758)	14.060(3.410)

*Note*. Numbers represent means. Numbers in parenthesis are standard errors of the mean. CON  =  not handled and group reared, EHLP  =  handled and group reared, IND  =  not handled and individually reared, EHLP + IND  =  handled and individually reared.

### Percentage of time spent in the open quadrants

**Figure 1:**
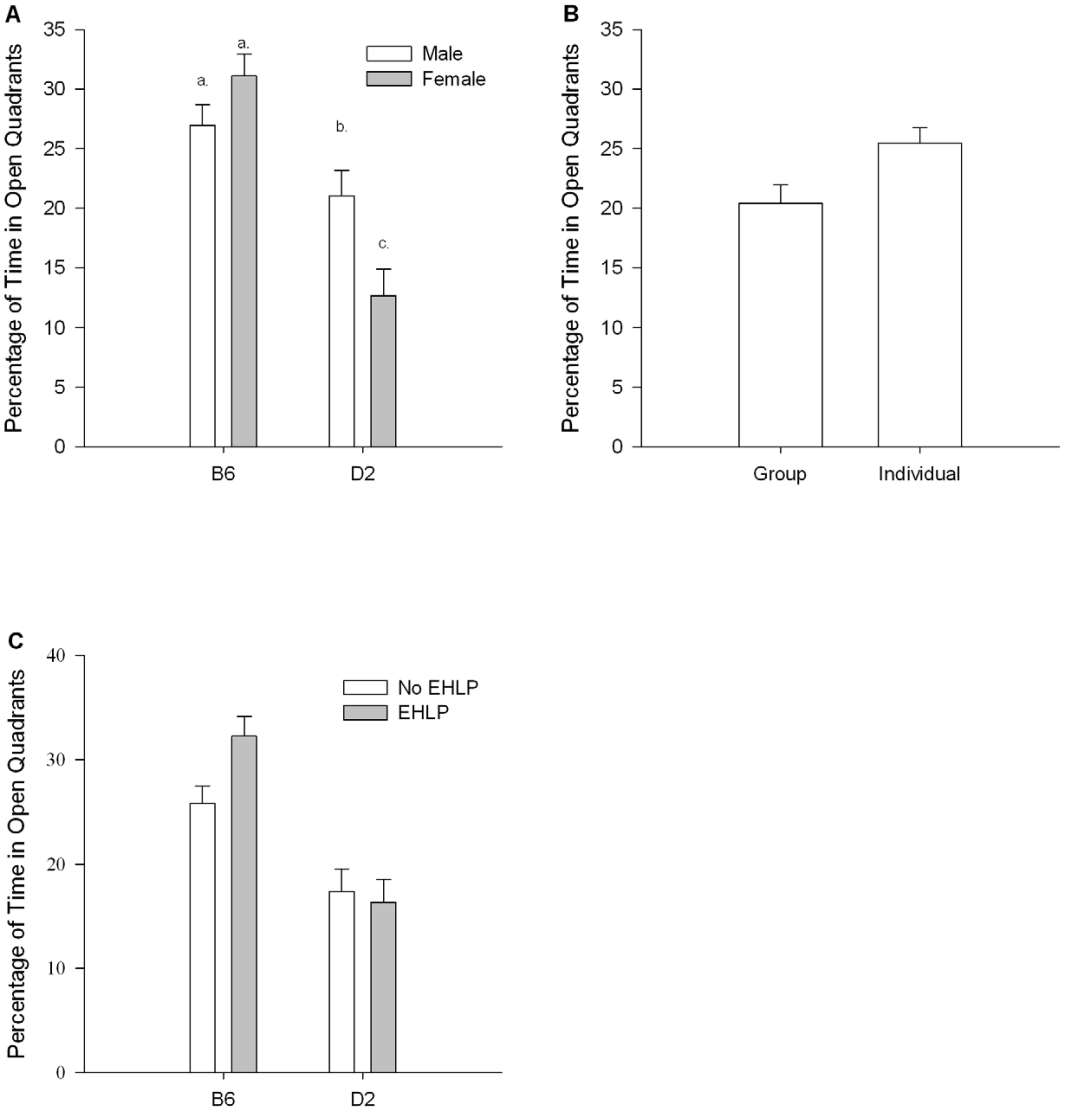
Percentage of Time in Open Quadrants of Elevated Zero-Maze. Data are presented as means ± *SEM*. **A**. Sex x strain interaction. Groups that do not share a common lowercase letter are significantly different at the level, *p*<.05. **B**. Main effect of housing, *p*<.05. **C**. Interaction of Strain x Handling. The effect did not quite reach statistical significance, *p* = .065. B6  =  C57BL/6J, D2  =  DBA/2J, Group  =  Group Housed, Individual  =  Individually Housed, No EHLP  =  undisturbed other than routine animal care, EHLP  =  Early Handling Like Procedure.

Means and standard errors are presented in Table 3. ANOVA revealed a significant effect of strain, *F* (1, 95) = 36.740, *p*<.001, with B6 mice spending more time in the open quadrants than their D2 counterparts. ANOVA failed to detect significant effects of sex or handling, *F* (1, 95) = 1.082, *p* = .301, and *F* (1, 95) = 1.853, *p* = .177, respectively. However, a significant effect of housing was found, *F* (1, 95) = 6.228, *p* = .014. (See Figure 1.) Group-housed animals spent less time in the open quadrants than individually-housed animals. ANOVA also revealed a significant interaction between strain and sex, *F* (1, 95) = 9.719, *p* = .002. (See Figure 1.) While B6 males and females did not differ significantly, D2 males spent more time in the open quadrants than did D2 females. Collectively, B6 males and females spent significantly more time in the open than did their respective D2 counterparts. ANOVA revealed a trend toward a strain x handling interaction; however, the interaction did not reach statistical significance, *F* (1, 95) = 3.475, *p* = .065. (See Figure 1.) B6, but not D2 mice that underwent the EHLP spent more time in the open than those that did not undergo handling. Handled and non-handled B6 mice spent more time in the open than D2 mice in these same groups. ANOVA failed to detect any other significant interactions (*p* >.10 for all).

**Table 3:** Means and standard errors for percentage of time spent in open quadrants.

	B6		D2	
Treatment	Male	Female	Male	Female
CON	21.330(3.503)	24.504(3.154)	17.408(7.020)	14.433(4.360)
EHLP	25.078(3.368)	29.807(2.884)	19.137(3.356)	11.711(2.742)
IND	23.860(3.357)	33.376(2.010)	24.770(5.747)	12.816(4.205)
EHLP + IND	37.403(1.760)	36.635(2.312)	22.760(3.566)	11.792(3.793)

*Note*. Numbers represent means. Numbers in parenthesis are standard errors of the mean. CON  =  not handled and group reared, EHLP  =  handled and group reared, IND  =  not handled and individually reared, EHLP + IND  =  handled and individually reared.

### Activity in the closed quadrants

Data for activity are provided in Table 4. ANOVA failed to detect any significant effects of: strain, sex, handling, or housing (*p*>.10, for all). However, ANOVA did reveal a significant interaction between strain and sex, *F* (1, 95) = 8.938, *p* = .004. Analysis of simple effects revealed that, while B6 and D2 males did not differ from one another, B6 females were more active than both B6 males and D2 females, but D2 males were more active than D2 females. There were no other significant interactions (*p*>.10, for all).

**Table 4:** Means and standard errors for activity in the closed quadrants.

	B6		D2	
Treatment	Male	Female	Male	Female
CON	2.131(.216)	2.525(.125)	2.289(.172)	2.071(.180)
EHLP	2.268(.088)	2.395(.142)	2.371(.120)	2.127(.094)
IND	1.877(.139)	2.379(.154)	2.352(.109)	2.023(.133)
EHLP + IND	2.421(.136)	2.379(.109)	2.185(.150)	2.060(.160)

*Note*. Numbers represent means. Numbers in parenthesis are standard errors of the mean. Activity is calculated as beam breaks per second spent in the closed quadrants. CON  =  not handled and group reared, EHLP  =  handled and group reared, IND  =  not handled and individually reared, EHLP + IND  =  handled and individually reared.

## Discussion

The purpose of this experiment was to characterize the effects of early-life manipulations (EHLP and individual-housing) on adult anxiety-like behavior in the EZM. Interestingly, although individual-housing increased the latency to enter an open quadrant, this manipulation resulted in anxiolytic-like behavior (increased time in the open quadrants). However, this effect was small, *d* = .224, and largely driven by the effects of individual-housing in B6 mice although the interaction was not significant. IND B6 mice spent about 33% of the time in open quadrants compared to 18% by their D2 counterparts (B6: IND 32.8±1.9 vs. CON: 25.2±1.7; D2: IND 18.0±1.8 vs. CON: 15.7±2.5). Similarly, others have reported anxiolytic-like effects of individual-housing on some behaviors in the EPM (i.e. percentage open time, [15], [16]) but not others (i.e. those activity-related [12], [13]). In contrast to our findings, it has been reported that individual-housing decreases latency to enter an open arm of the plus-maze [16]. It is likely that a number of experimental and experiential factors contribute to differences in findings, including differences in maze types and the fact that the latency variable is often difficult to interpret [38]. That we did not extend our studies to include other anxiety-related tasks is a notable limitation. For example, increased defecation in the hole-board and light/dark tests suggests an anxiogenic effect of individual-housing [15], [16]. Individual-housing has also been shown to increase activity in the open field [16], hole-board, and EPM [15]. In contrast, decreases in home cage activity have been reported following individual-housing [39]. Certain behavioral tasks may be more or less sensitive to the types of manipulations we carried out.

Contrary to expectations, we also observed a strain-specific trend of anxiolytic effects associated with the EHLP. Although this effect fell short of statistical significance, a comparison of effect sizes of the EHLP effects reveals that the manipulation had a much larger effect in B6 mice than in D2 mice, *d* = .614 and *d* = .084 respectively. This interaction is worthy of further investigation considering the large difference in response. Others have found early-handling (10–15 min separation) to have an anxiolytic effect [34], [40], or no effect at all [41]. However, MS (180 min separation) in mice has been reported to increase anxiety-like behavior [41]–[43] or be without effect, with specific findings often depending on sex, strain, and task [41]–[43]. Our results are most similar to those reported for early-handling (*vide supra*), particularly as our 30 minute separation period is closer to the 15 minute separation used in early-handling paradigms, than the 180 minutes used in MS paradigms. However, our findings are differentiated from those of prototypical MS and early-handling paradigms by the developmental context of the manipulation. The developmental window during which we carried out the EHLP (PND 12–20, after the SHRP) is characterized by increased emotionality and exaggerated responses to stimuli [44], [45]. During this period, the animal may also be particularly susceptible to environmental manipulations, as the maturation of several neural circuits important to anxiety-like behaviors is also occurring [10]. Only a limited number of studies have examined the effects of environmental manipulations occurring after the SHRP in pre-weanling mice. It has been reported that a repeated daily 30 min separation at PND 10–21 did not result in differences in neurobehavioral development, but did increase the latency for pups to return to their nest in the homing test [8]; however, behavioral testing of adult animals was not performed. When a single 24 hr separation is carried out in mice on PND 9 or 12, there is no effect on behaviors in the open field [46], [47], but when separation occurred at PND 9, an anxiolytic-like effect in the EPM was observed [46].

It has been suggested that the effects of handling may be due to the animal's increased habituation to novelty [19], [48]. B6 mice generally habituate more readily than D2 mice to repeated exposures to the open field [49], [50], hole-board [16], and EZM [51] tests. It is difficult to determine the extent to which any differences in habituation to novelty influenced our results, particularly as the strain by treatment interaction did not reach statistical significance. However, our results suggest that handling procedures performed after the SHRP may be of value in investigations of gene-environment effects on anxiety-like behavior.

Our primary goal here was to characterize the behavioral effects of these manipulations, but we note the absence of physiological measures. Few studies, however, have examined the physiological effects of environmental manipulations occurring after the SHRP. In rats, MS (360 min) from PND 15–21 resulted in increased plasma CORT levels both during the MS procedure and in adulthood [52]. Reasonable extensions of the present study would include varying the EHLP manipulation (e.g. longer periods of separation) as well as measuring plasma CORT following the manipulation as well as in adulthood. Furthermore, as the effects of typical MS and early-handling procedures are related to maternal behavior [53], it would be valuable to examine how manipulations applied after the SHRP affect maternal behavior. We chose B6 and D2 mice because of known difference in a number of behaviors/measures relevant to the present study [25]–[28]; however, testing other inbred mouse strains would be a good first step toward exploiting the wealth of murine genetic models. Although our findings are rather modest, such studies are useful first steps in understanding the complex gene-environment interactions that characterize anxiety disorders.

We were also interested in ascertaining any interaction between the early manipulation (EHLP) and the post-weaning manipulation (individual-housing) on anxiety-related behavior in the zero-maze. We did not observe any significant interactions between these manipulations. It has been shown that a 60 s handling procedure performed every 48 hrs at PND 3–21 interacted with individual-housing in mice to affect physiological measures, but interactions between these factors were not reported for the behavioral tests performed [19]. Recently, MS (180 min) at PND 2–14 followed by individual-housing increased ethanol preference in adult B6 females but not males [24]. Such studies highlight the merit of examining such interactions as well as how these interactions influence not only anxiety-related behaviors, but conditions like drug-abuse that are often co-morbidly presented.

Our findings demonstrate that early experiences influence adult behavioral phenotypes, although some (i.e. individual-housing) to a greater extent than others (EHLP). Future investigations using additional inbred strains and behavioral tests would be useful in elucidating the influence of early-life events on pathological states like anxiety in the adult organism.
